# Bibliometric analyses of climate psychology: critical psychology and climate justice perspectives

**DOI:** 10.3389/fpsyg.2025.1520937

**Published:** 2025-02-13

**Authors:** Gulnaz Anjum, Mudassar Aziz

**Affiliations:** ^1^Department of Psychology, University of Oslo, Oslo, Norway; ^2^Department of Psychology, University of Limerick, Limerick, Ireland

**Keywords:** bibliometric analysis, climate psychology, critical psychology, climate justice, eco-anxiety, research equity, mental health, climate change

## Abstract

The psychology of climate change has become a critical area of research, exploring the intersection between human behavior, psychological wellbeing, and environmental sustainability. This paper presents a bibliometric analysis to explore the interdisciplinary field of psychology and climate change, covering research from 01 January 1995 to 15 August 2024. Using 3,087 academic publications from the Web of Science and employing VOSviewer and BiblioMatrix for network analysis, we dissect the evolution, key contributors, and central themes within this domain. Our analysis identifies leading authors, institutions, and nations, alongside the collaboration networks underlying the field’s growth. Thematic clustering of these networks highlights dominant topics such as pro-environmental behavior, sustainability, mental health, eco-anxiety, and risk perception. We utilize visual mappings of co-authorship and bibliographic relationships to illustrate the dynamic interaction among researchers and their topics. By framing our findings through the lens of climate justice and critical psychology, we advocate for a research paradigm that challenges systemic barriers to climate justice, emphasizing the necessity for equitable and action-oriented psychological research to guide climate-related policy and public engagement.

## Introduction

The psychology of climate change has emerged as a dynamic and multifaceted field, reflecting the urgency of addressing the environmental, social, and psychological challenges posed by the climate crisis. This interdisciplinary area of research seeks to explore how individuals and communities perceive, experience, and respond to the impacts of climate change, emphasizing the cognitive, emotional, and behavioral dimensions of human-environment interactions. As climate change intensifies, with increasingly frequent and severe consequences globally, understanding the psychological mechanisms underlying public engagement, pro-environmental behavior, and adaptation strategies becomes critical. Additionally, the ethical and justice-oriented aspects of climate action have gained prominence, necessitating a focus on systemic inequities and the disproportionate burdens placed on vulnerable populations (Aziz and Anjum, 2024). This paper utilizes a bibliometric analysis to map the global research landscape of the psychology of climate change and climate justice, aiming to pinpoint key contributions, thematic areas, and collaborative networks that have shaped this field.

### Key themes and approaches in climate psychology

This review adopts a bibliometric analysis approach to map the evolution and key themes of climate psychology through the lens of critical psychology and climate justice. The key literature on the topic can be structured around four key thematic areas. First, the role of psychological constructs, particularly psychological distance, is discussed to highlight how individuals perceive and engage with climate change. Second, effective communication strategies and the framing of climate change issues are analyzed to explore how tailored approaches can enhance public understanding and motivate collective action. Third, the mental health impacts of climate change are examined, emphasizing the direct and indirect psychological toll on individuals and communities. Finally, the systemic barriers to equitable climate governance are addressed, underscoring the importance of integrating climate justice into psychological research. By synthesizing these interconnected dimensions, the review aims to highlight the critical role of psychology in addressing the climate crisis and advocating for inclusive, justice-oriented approaches to climate action.

#### Psychological constructs

The crisis of climate change poses unprecedented challenges to global ecosystems, human livelihoods, and social structures, necessitating a multidisciplinary approach to understand the landscape of contemporary approaches. Building on the interdisciplinary nature of this field, the psychology of climate change is understood as a multidimensional domain that examines how individuals and communities interpret, experience, and respond to the multifaceted challenges posed by climate change (Anjum and Fraser, 2021).

The urgency of addressing climate change is underscored by the Intergovernmental Panel on Climate Change (IPCC), which highlights the critical role of human behavior and societal transformation in combating environmental degradation (IPCC, 2021). Psychological research contributes to this by examining the cognitive, emotional, and social factors influencing environmental attitudes, behaviors, and policy support (Clayton and Manning, 2018; Gifford, 2011; Swim et al., 2011). Furthermore, the concept of climate justice introduces an ethical dimension to the climate discourse, emphasizing the disproportionate impact of climate change on vulnerable populations and the need for equitable mitigation and adaptation strategies (Schlosberg, 2013; Caney, 2014). The integration of psychology into climate change research is not only essential for understanding the human dimensions of environmental change but also for devising effective interventions to foster pro-environmental behavior and resilience in the face of climate threats (Clayton et al., 2021; Reser and Swim, 2011). Studies have explored various psychological aspects, including risk perception, environmental identity, and the efficacy of communication strategies in enhancing public engagement with climate action (van der Linden, 2015; Ojala, 2012).

A key construct in understanding climate change perception is psychological distance, which influences pro-environmental behaviors. The research presents mixed results: some studies indicate that perceiving climate change as a proximal threat increases mitigation and adaptation efforts, while others show no significant effect. This inconsistency underscores the need for further investigation into psychological variables mediating this relationship (Maiella et al., 2020). Furthermore, these debates reveal the importance of considering individual and cultural variability in understanding how climate change is perceived and acted upon.

#### Climate change communication: frames and challenges

Effective communication about climate change is central to addressing its challenges. The literature suggests a focus on pro-environmental behavior determinants, with psychologists urged to deepen theoretical understandings of governance through ecological and systemic perspectives (Freschi et al., 2023). Communication strategies often examine frames such as public health, economic development, and environmental impact, with mixed results regarding their efficacy. Tailoring strategies to audience-specific contexts is essential for enhancing public understanding and engagement (Badullovich et al., 2020).

Despite these efforts, there remains an ongoing debate about how to best communicate the urgency and equity dimensions of climate change. Communication often prioritizes individual behavior change or public opinion influence but fails to address systemic issues like unequal distribution of climate impacts. Critical psychology advocates for strategies that empower communities and mobilize collective action against systemic injustices, ensuring that the responsibility of high-emitting entities is not overlooked. By framing communication through a justice-oriented lens, the psychological dimensions of governance and public engagement can be more effectively addressed.

#### Mental health impacts of climate change

Climate change affects mental health directly through acute events (e.g., hurricanes, floods) and indirectly through chronic stresses (e.g., heat, drought). These impacts manifest as increased risks of post-traumatic stress disorder, anxiety disorders, depression, and other mental health issues. Vulnerable groups, particularly women in low-and middle-income countries (LMICs), face exacerbated conditions, emphasizing the need for targeted interventions (Cianconi et al., 2020; Aziz and Anjum, 2024). Recent policy perspectives highlight the disproportionate impact of climate change on women, particularly pregnant women and newborns, advocating for transformative strategies to enhance resilience. Such strategies include dismantling discriminatory socio-cultural norms, strengthening health systems, and integrating digital health literacy, political empowerment, and sexual and reproductive health rights into climate adaptation efforts. These multi-sectoral approaches, tailored to address the specific health challenges faced by women in LMICs, underscore the importance of fostering community-based interventions and incorporating gender-sensitive perspectives into climate mitigation strategies (Aziz and Anjum, 2024).

Research has also shown that structural violence and injustice shape these mental health outcomes. Marginalized populations experience climate change as an immediate reality intertwined with environmental justice, resource access, and historical inequities. Frameworks like psychological distance often fail to incorporate these lived experiences, inadvertently marginalizing narratives of justice and ignoring the pressing needs of these communities (Maiella et al., 2020). A justice-focused perspective necessitates systemic changes addressing the root causes of both mental health crises and climate change (Clayton et al., 2021; Doherty and Clayton, 2011).

#### Critical psychology lens to the major narratives in climate psychology

Climate justice emphasizes the equitable distribution of climate change impacts and the moral responsibility to protect vulnerable populations disproportionately affected by climate crises (Ogunbode et al., 2024). Critical psychology enhances this discourse by examining how power structures and systemic inequities influence perceptions of and responses to climate justice, advocating for justice-oriented approaches that bridge individual beliefs and collective action. Critical psychology, with its emphasis on power dynamics, social justice, and the broader socio-political context, offers a unique perspective on the psychological aspects of climate change, highlighting how mainstream psychology might overlook the systemic injustices and disparities that climate change exacerbates (Prilleltensky, 2012). The debate on individual perception and behavioral response to climate change primarily focuses on cognitive and motivational factors influencing personal actions toward mitigation or adaptation.

From a critical psychology perspective, debates on the psychology of climate change often fail to account for the structural inequalities that shape these perceptions and behaviors. Discussions rarely address how marginalized communities perceive climate change through the lens of ongoing social and environmental injustices or how systemic barriers limit their capacity to respond (Anjum and Fraser, 2021). Without incorporating these aspects, we risk neglecting how power imbalances and social determinants influence climate-related behaviors and perceptions, sidelining the importance of climate justice in shaping equitable responses to climate change.

The dominance of the Global North in psychological research further underscores these systemic disparities. Research agendas, funding structures, and publication outputs are disproportionately controlled by institutions in WEIRD (Western, Educated, Industrialized, Rich, and Democratic) countries, particularly the United States and Europe (Arnett, 2008). This imbalance not only restricts the diversity of theoretical and methodological approaches but also marginalizes the contributions of Global South researchers, who bring critical local insights to the field. Structural barriers, such as unequal access to funding and representation on editorial boards, further hinder their participation in global scholarly discourse (Patel, 2014; Liu et al., 2024). Bridging these gaps is essential for developing a truly inclusive understanding of climate psychology that reflects the lived experiences of underrepresented populations.

Research indicates diversity in research designs, outcome variables, and theoretical perspectives on climate change within psychology. However, gaps exist, such as a weak presence of non-Western perspectives, a lack of cross-cultural comparisons, and an overemphasis on intrapersonal processes (see Anjum and Aziz, 2024). Future research should broaden geographical and demographic representation and examine outcomes beyond mitigation behavior, adopting more “social” and “equity” theoretical perspectives (Tam et al., 2021; Anjum and Aziz, 2024).

The mental health impacts of climate change also highlight the limitations of conventional frameworks. While the psychological toll of climate change, including anxiety, depression, and PTSD, is widely acknowledged, these discussions often fail to connect individual mental health outcomes to the broader socio-political contexts that exacerbate them. Structural violence, resource inequities, and historical injustices significantly shape these mental health vulnerabilities, particularly for marginalized groups (Clayton et al., 2021; Doherty and Clayton, 2011). A critical psychology lens challenges the tendency to isolate mental health from its systemic roots, emphasizing the importance of justice-oriented approaches that address both individual wellbeing and structural inequities.

Furthermore, psychological constructs such as psychological distance often neglect the immediacy of climate change for vulnerable populations. For many, climate change is not a distant abstraction, but a present and tangible reality shaped by environmental injustices and systemic inequities (Maiella et al., 2020). Critical psychology calls for reframing these constructs to incorporate the lived experiences of communities facing the brunt of climate impacts. This shift ensures that justice narratives are central to understanding and addressing public engagement with climate change.

Effective communication strategies offer another critical area where a justice lens is essential. Traditional communication often emphasizes individual behavior change or public opinion shifts while failing to address systemic injustices. Justice-oriented communication reframes climate messaging to highlight the unequal distribution of climate impacts and the disproportionate responsibility of high-emitting countries and corporations (Badullovich et al., 2020). Empowering communities to challenge systemic inequities through collective action can significantly enhance the impact of climate communication efforts.

In climate governance, psychology’s role extends beyond influencing individual and group behaviors to addressing systemic barriers that perpetuate inequities. A critical psychology perspective advocates for examining who benefits and who is disadvantaged by current climate policies, ensuring that governance frameworks prioritize the needs of the most vulnerable populations. By integrating climate justice into governance strategies, psychological research and practice can contribute to equitable and sustainable solutions that challenge the status quo of environmental and social inequalities (Schlosberg, 2013; Caney, 2014).

### Current research

The aim of this study is to conduct a bibliometric analysis to explore the global landscape of climate psychology research, identify key contributors, themes, and gaps, and critically assess the dominance of Global North perspectives using a climate justice and critical psychology lens. The current study used bibliometric analysis for this exploration. Bibliometric analyses serve as powerful tools for examining the structure, prevalence, and dynamics of scientific research. Therefore, it has the potential for offering insights into the trends in a field, i.e., the most influential studies, authors, and institutions driving the field (Aria and Cuccurullo, 2017). By applying bibliometric techniques to the topics of climate change and climate justice within psychology, this study aims to provide a comprehensive overview of the field’s intellectual landscape, identifying core themes, emerging trends, and gaps in the literature. This paper is structured as follows: First, we outline the methodology employed for the bibliometric analysis, including data collection, software tools, and analytical techniques. Next, we present the results of the analysis, highlighting key findings related to publication trends, thematic clusters, and research networks. Finally, we discuss the implications of these findings for future research directions and policy interventions, emphasizing the critical role of psychological insights in advancing climate justice and sustainability goals.

## Methodology

This study employs bibliometric analysis to systematically review and analyze the body of literature on the psychology of climate change. To capture the comprehensive landscape of research in this field, we refined our search strategy to include terms related to “climate change” and “psychology,” encompassing a broad range of psychological aspects (e.g., attitudes, behaviors, mental health) related to climate change. This approach is informed by prior literature retrieval methods that emphasize the importance of encompassing both direct and peripheral dimensions of research topics to fully understand their scope and impact (Aria and Cuccurullo, 2017).

### Objectives of the research

The primary objectives of this research are:

1. To map the global research landscape of the psychology of climate change, identifying key contributions, thematic areas, and collaborative networks that have shaped this field from January 1995 to August 2024.
2. To identify leading authors, institutions, and nations contributing to the research on climate change psychology. This includes examining their roles and influence within the collaborative networks that drive this field’s growth.
3. To employ thematic clustering of research networks to highlight dominant topics such as pro-environmental behavior, sustainability, mental health, eco-anxiety, and risk perception. This includes analyzing how these themes have evolved over time and identifying emerging research frontiers.
4. To utilize visual mappings of co-authorship and bibliographic relationships to illustrate the dynamic interactions among researchers and their topics. This helps in understanding the collaborative nature of research and the interconnectedness of different research themes.
5. To frame the findings within the perspectives of critical psychology. This involves advocating for a research paradigm that challenges systemic barriers to climate action and emphasizes the necessity for equitable and action-oriented psychological research to guide climate-related policy and public engagement.

### Literature retrieval

The expansion of our research literature retrieval focused on the intersection of climate change and psychology. We utilized the Web of Science Core Collection (WoSCC) to extract bibliometric data, specifically targeting literature that combines aspects of psychology with climate change. The search spanned over the period from 1995 to 20th February 2024 were considered, resulting in a total of 3,456 documents. This research was update on 15 August 2024. This corpus encompassed various document types, including articles, editorial materials, letters, meeting abstracts, and reviews. All retrieved papers from the WoSCC, encompassing titles, keywords, author information, abstracts, and references, were downloaded and stored in Bibtex file format. Additional details are provided at OSF.^1^

### Inclusion and exclusion criteria

The inclusion criteria encompassed peer-reviewed articles written in English, as these represent the core of scientific discourse that is accessible to the international research community. Exclusion criteria were applied to filter out non-relevant document types such as editorials, conference proceedings, and book reviews, focusing the analysis on original research articles and review papers that contribute substantive empirical and theoretical insights to the field. Studies that were primarily focused on the work climate and the school climate within psychology were excluded from the analysis. Moreover, literature focused on sports climate in psychology was not included in any analysis.

The search was conducted across the titles, abstracts, and keywords of articles indexed in the Web of Science Core Collection (WoSCC). The WoSCC was selected as the primary database due to its extensive coverage of high-quality scientific literature across biomedical, natural, social sciences, and its recognition as one of the most comprehensive databases for bibliometric analyses (Wu et al., 2021) This database not only provides access to a vast array of publications but also includes detailed citation information, allowing for a comprehensive analysis of the influence and evolution of research within the field.

### Data analysis

To ensure the analysis reflects the most current trends in research, the search was limited to articles published up to the most recent full calendar year. After extracting the bibliometric data, the study utilized VOSviewer (Version 1.6.16, Leiden University, the Netherlands) software for network analysis, facilitating the visualization of co-authorships, bibliographic couplings, and co-citation networks. This analytical approach allows for the identification of key themes, influential authors, and institutions, as well as the tracing of the thematic evolution and interconnections within the research landscape of the psychology of climate change.

**Figure 1:**
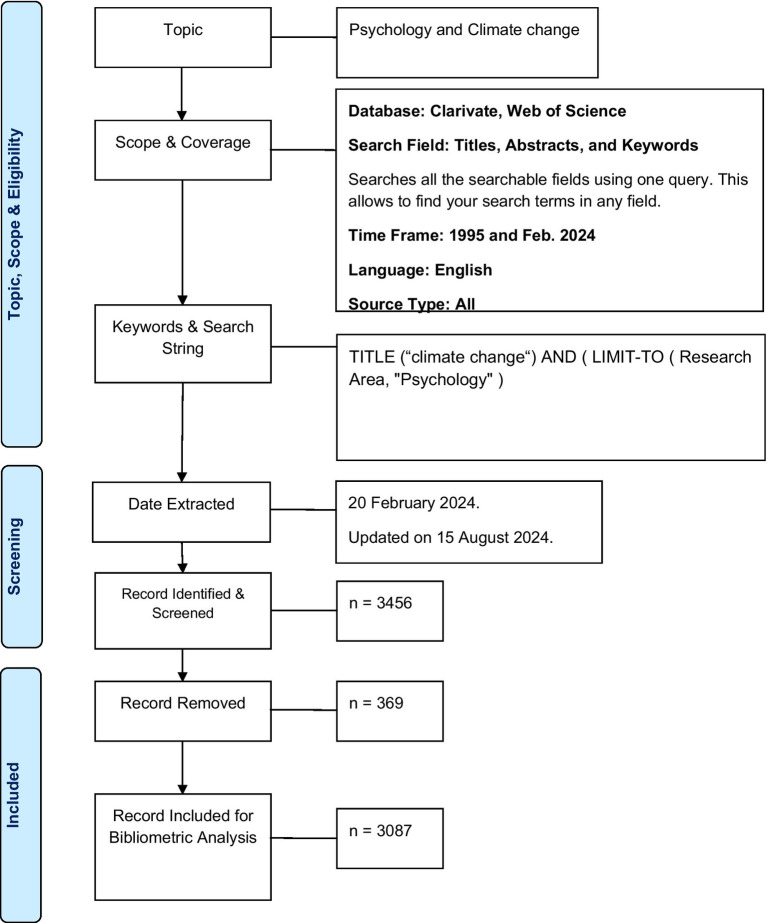
PRISMA diagram: flow diagram of the included papers.

We employed a bibliometric analysis approach to assess the selected corpus of 3,087 articles on the psychology of climate change. The Bibtex files were imported into the Biblioshiny application using R software (version 4.0.2), RStudio software (version 1.3.959), and the bibliometrix R package,^2^ converting the original data into a data frame set. Subsequently, these files were imported into Microsoft Excel 2019 for additional data processing. To ensure the reliability of the results, two researchers independently conducted the literature selection, data extraction, and analysis. The extracted data encompassed general information such as the annual number of publications, citation frequency, countries of origin, authors, journals, and institutions. The quality of the authors’ publications was assessed based on metrics including the number of publications, citations in the research area, and the H-index value. Data analysis and visualization were conducted using VOSviewer, which facilitated the creation of network visualization maps to examine collaborative relationships between countries/regions, institutions, and authors of highly cited references. Additionally, VOSviewer’s co-occurrence analysis feature was utilized to classify keywords with high co-occurrence frequencies into several clusters, coloring them by time course to identify research hotspots and trends. Details of the process of literature retrieval and assessment are given in the PRISMA diagram presented in Figure 1.

## Results

### Publication output and temporal trend

**Figure 2:**
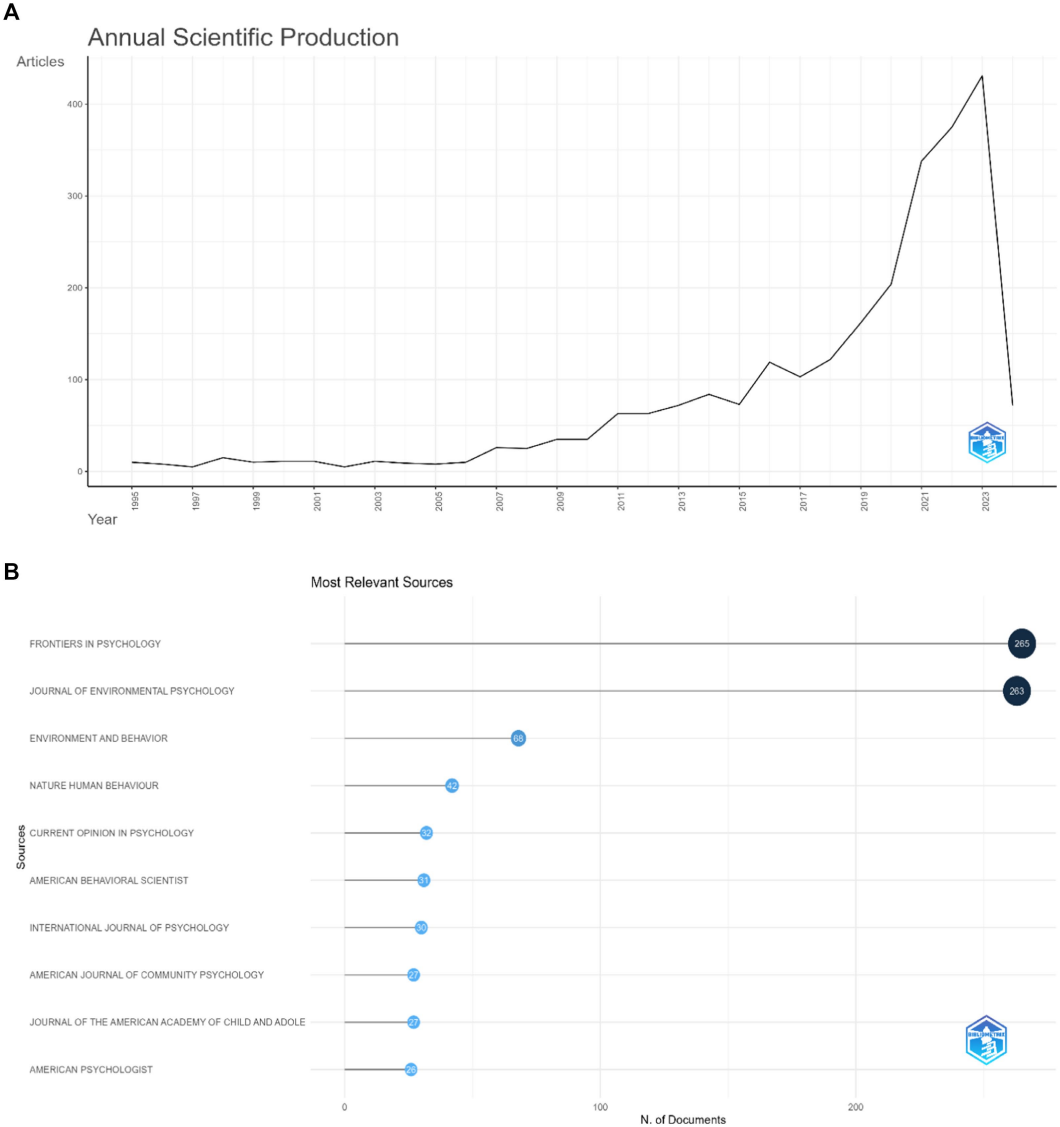
**(A)** Global trend of annual publications from 1995 to 2024. **(B)** Most relevant sources.

According to the above retrieval methods and data processing, a total of 3,087 publications were obtained from the WoSCC, which were published from 1995 to to-date (20th Feb 2024). Among them, there were 2,573 papers (83.34%), 183 reviews (5.92.92%), 122 editorial materials (3.95%), 86 meeting abstracts (2.78%), and 15 letters (0.48%) 85 proceeding papers (2.75%), 13 Book reviews (0.42%), 10 book chapters (0.32%). Since 1995, the publications of related literature on “Climate Change” and “psychology” showed a fluctuating upward trend, reaching two peaks in 2016 and 2023, and 2023 was the most prolific year for publications. These results are shown in Figure 2A.

### Analysis of leading journals (Frontiers in Psychology)

Figure 2B presents the top 10 most popular journals contributing to articles on climate change and psychological topics. Frontiers in Psychology was the leading journal, publishing the most papers (265 articles), followed by Journal of Environmental Psychology (263 articles), Environment and Behavior (68 articles), Nature Human Behavior (42 articles), Current Opinions in Psychology (32 articles,), American Behavioral Scientist (31 articles), International Journal of Psychology (31 articles), American Journal of Community Psychology (27 articles), Journal of American Academy of Child and Adolescents (27 articles, England), American Psychologist (26 articles). The graphs indicate trends in producing scholarly literature and citation practices over time, which is essential for understanding the development of research areas and their relative influence or impact within psychology as a field.

**Figure 3:**
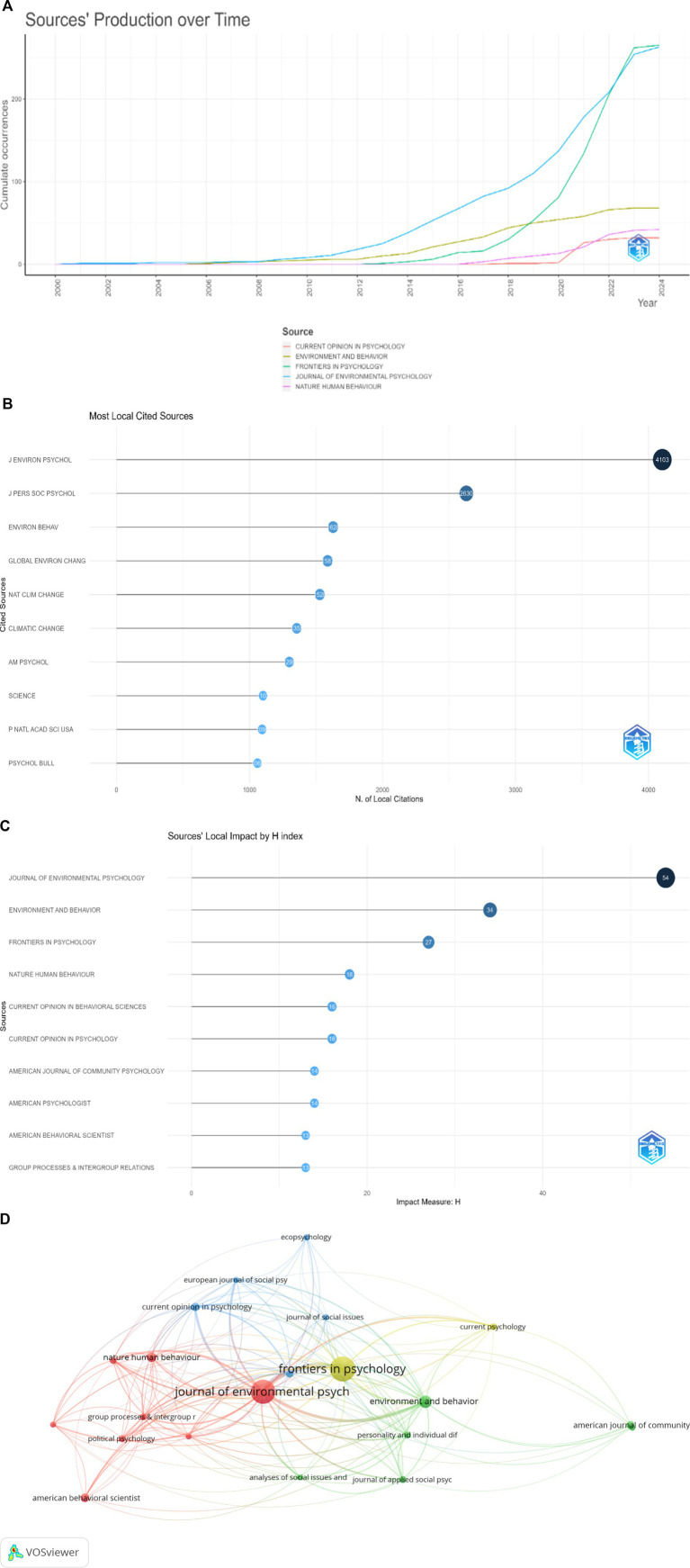
**(A)** Journal’s production over time. **(B)** Most locally cited journals. **(C)** Journal’s local impact by H-index. **(D)** Citation map of journals on psychology and climate change. Each node represents a journal, and node size indicates the number of publications. The connection between the nodes represents a citation relationship, and the thickness of the lines indicates citation strength.

It’s clear from the visualizations (see Figure 3A) that the psychology of climate change, for instance, has seen a significant increase in scholarly output over time, as evidenced by the increasing trend lines in the cumulative occurrences graph. Notably, the journals “Frontiers in Psychology” and “Journal of Environmental Psychology” have shown notable growth in the number of documents produced, indicating a heightened scholarly focus on the psychological dimensions of human interaction with climate change.

Over the years, the production of scholarly articles in these fields has grown substantially. Notably, “Frontiers in Psychology” has seen a steady trajectory of growth, evidenced by its prominent curve in the sources’ production over time graph (Figure 3A). This trend signifies the journal’s expanding role in propelling the knowledge base in psychology, particularly from a bio-psycho-social perspective.

The data on the most locally cited sources reveal the central position of “Journal of Environmental Psychology” within the academic community, amassing 4,103 local citations (Figure 3B). The citation frequency of this journal not only reflects its contributions to the field. High citation counts are often associated with journals that have not only a substantial H-index but also a significant impact factor. Furthermore, when examining citation patterns, the data indicates that the “Journal of Environmental Psychology” holds a prominent position in terms of local citations, suggesting its considerable influence within the field. This might be due to its longer establishment, since 1981.

Figure 3C depicts H-index that is a metric that measures both the productivity and citation impact of the publications by a scholar or in this case, by a journal. It reflects the number of articles (H) that have received at least H citations over time. It is often used as an indicator of the significance and impact of a researcher’s cumulative research contributions. In this context, the “Journal of Environmental Psychology” has the highest H-index of 54, indicating its substantial influence and the pivotal role it plays in the field. It’s followed by “Environment and Behavior” and “Frontiers in Psychology,” with H-index scores of 34 and 27, respectively. These journals are recognized for their significant contributions to the climate change in their fields. “Nature Human Behaviour.” Moreover, the two series from “Current Opinion,” namely “Behavioral Sciences” and “Psychology,” exhibit H-index scores of 18 and 16 each. The journals “American Journal of Community Psychology,” “American Psychologist,” and “American Behavioral Scientist” have similar H-index scores ranging from 13 to 14. “Group Processes & Intergroup Relations” also appears on the graph with an H-index of 13, closing the list of the depicted journals. It suggests that while this journal has a specific focus, it maintains relevance and is cited consistently in related research.

Figure 3D is a network visualization map generated by VOSviewer. This type of visualization represents the relationships between various scholarly journals based on citation and co-citation. In the network, each node (circle) represents a different journal, and the lines (edges) between the nodes signify the type of relationship analyzed, i.e., their co-citation frequency. The size of the nodes correlates with the magnitude of the metric being measured, such as the number of citations or papers, while the thickness of the edges between nodes may represent the strength of the relationship (e.g., how frequently two journals cite each other). Two journals in particular, “Journal of Environmental Psychology” and “Frontiers in Psychology,” appear prominently, which likely indicates their central role within the network, possibly reflecting higher citation counts, greater impact, or a pivotal position within the field’s research landscape. The various colors in the network could represent different clusters or groups within the field of psychology, which are formed based on similarities in research topics, methodologies, or interdisciplinary connections. For example, journals clustered together in the same color tend to publish research on similar themes or be part of a specific sub-discipline within psychology.

It’s also evident from the map that there is a web of connections between various journals, indicating an interconnected research field where findings and theories are frequently built upon and referenced across different publication outlets. This network visualization provides a macro-level view of the academic landscape of climate psychology, offering insights into which journals are most central to the field’s discourse and how they interrelate.

### Analysis of authors

**Figure 4:**
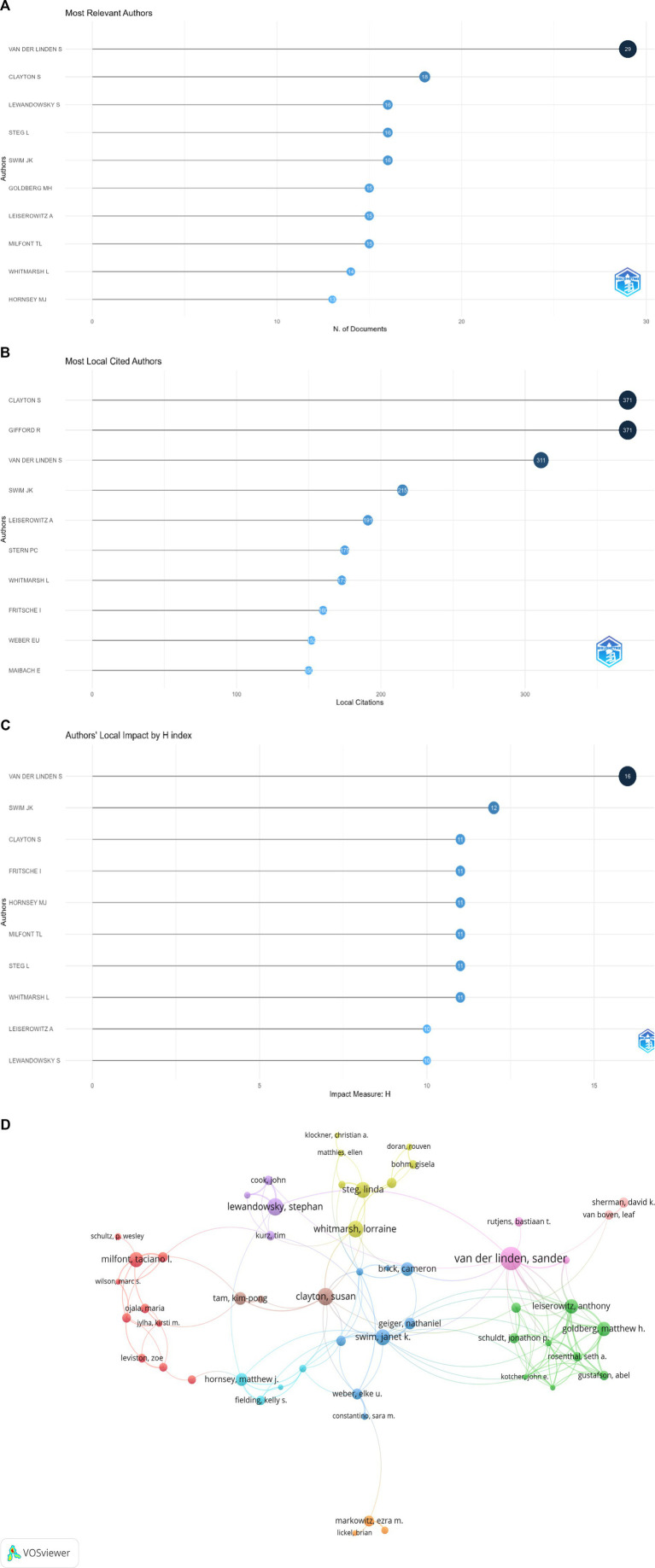
**(A)** Most relevant authors by number of documents. **(B)** Number of local citations each author has received. **(C)** Authors ranked by their H-index. **(D)** Network visualization: displaying the co-authorship network among authors.

Our analysis of author is shown in Figure 4A. It shows the ranking of authors by the number of documents they have contributed to the field. The x-axis represents the number of documents, and the y-axis lists the authors’ names with their initials. S. van der Linden is the most prolific author, with a count of 29 documents, followed by S. Clayton and S. Lewandowsky with 18 and 16 documents, respectively.

Figure 4B shows another bar graph showing the number of local citations each author has received. S. Clayton and R. Gifford are the most cited authors, each with 371 citations, which implies that their work is highly cited and considered important within the research community. Figure 4C illustrates authors ranked by their H-index. S. van der Linden has the highest H-index of 16, suggesting a strong influence and high-quality contributions. The graph shows other authors as well, with H-index scores ranging from 11 to 16.

Figure 4D is network visualization from a bibliometric tool VOSviewer, displaying the co-authorship network among authors. The size of the nodes corresponds to the number of documents. The lines between the nodes indicate co-authorship. The different colors can represent different clusters or research groups, possibly highlighting the authors’ focus areas or collaborative patterns. The central figures, such as van der Linden and Clayton, are likely key influencers in their networks, as indicated by their larger node size and numerous connections. These visualizations provide insight into the impact and productivity of authors in a specific research area. By analyzing the number of documents, citations, H-index, and collaborative networks, we can infer the central figures within the field and identify potential collaboration opportunities.

### Representation of institutions

To show which institutions are getting represented in the current landscape of research, the Table 1 showcases the top 10 universities with significant academic impact in their field. This is determined through their sum of citations and documents published. Leading the list is the University of Western Australia, which has amassed 3,384 citations from 27 documents, highlighting its research’s considerable influence. Following closely is the University of Queensland, with 3,023 citations from 35 documents, indicating a prolific output with a strong impact. The University of Victoria notably achieves a high citation count of 2,676 from just 15 documents, suggesting that its research is not only voluminous but also of remarkable significance. The University of Michigan presents a substantial 2,642 citations from 31 documents, pointing toward a robust and influential research environment. Yale University is next with 43 documents, receiving 1,987 citations. Cardiff University shows a significant citation count with 1,831 from 29 documents, George Mason University with 1,754 from 25 documents, and the University of Melbourne with 1,728 from 33 documents, each contributing substantially to the academic discourse. College of Wooster and Columbia University round out the top 10, with 1,685 and 1,611 citations from 18 and 32 documents, respectively, underlining their research’s substantial reach and impact within the academic community.

**Table 1:** Top 10 universities with significant academic impact in their field, as evidenced by their sum of citations and documents published.

Organization	Sum of citations	Sum of documents
University of Western Australia	3,384	27
University of Queensland	3,023	35
University of Victoria	2,676	15
University of Michigan	2,642	31
Yale University	1,987	43
Cardiff University	1,831	29
George Mason University	1,754	25
University of Melbourne	1,728	33
The College of Wooster	1,685	18
Columbia University	1,611	32
Penn State University	1,523	32
University of Exeter	1,340	22
NYU	1,320	26
University of Bristol	1,231	16
Oxford University	1,176	17
Stanford University	1,138	27
Princeton University	1,122	18
University of Rochester	1,104	5

**Figure 5:**
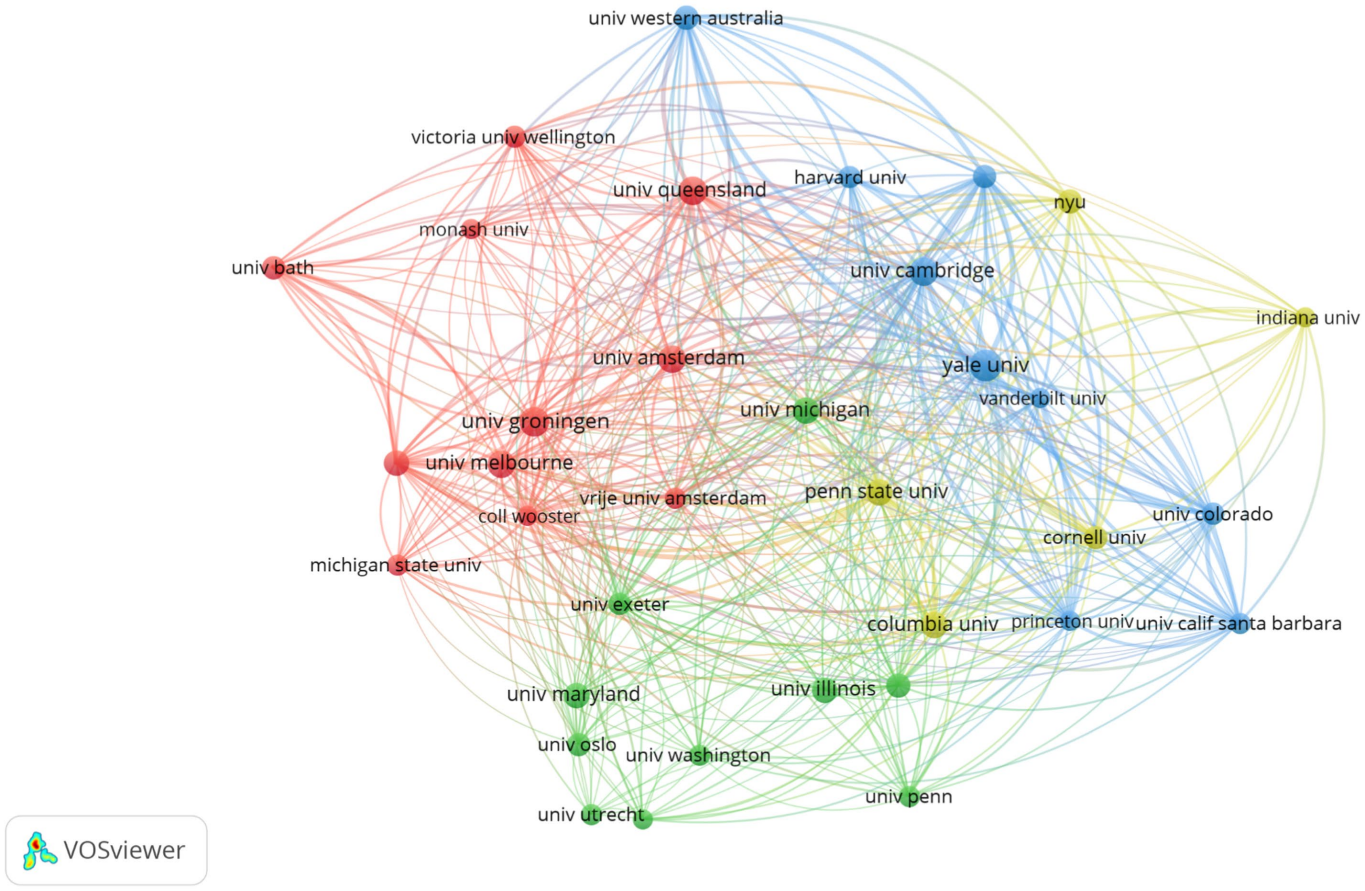
Clusters of universities, grouped by research collaboration.

Based on the network visualization provided, which is a bibliometric analysis of universities in a specific research context we can observe the visualization shown in Figure 5. It shows clusters of universities, grouped by research collaboration. There is a noticeable cluster involving the University of Melbourne, Griffith University, and other Australian universities, indicating a strong regional collaboration network. There are numerous interlinking lines that suggest cross-institutional collaborations or citations. The density and centrality of these connections can signal the extent to which research from these institutions is interwoven within the global academic community. The presence of universities from various parts of the world suggests a global network of research activities. For instance, we can see representation from North America, Europe, Asia, and Australia, indicating the international scope of research in the field. This type of network visualization helps to identify key research institutions and understand the collaborative dynamics within a specific academic field. It can be particularly useful for new researchers looking to find potential institutions for collaboration or for established researchers aiming to understand the impact and reach of their work.

### Analysis of leading countries

**Figure 6:**
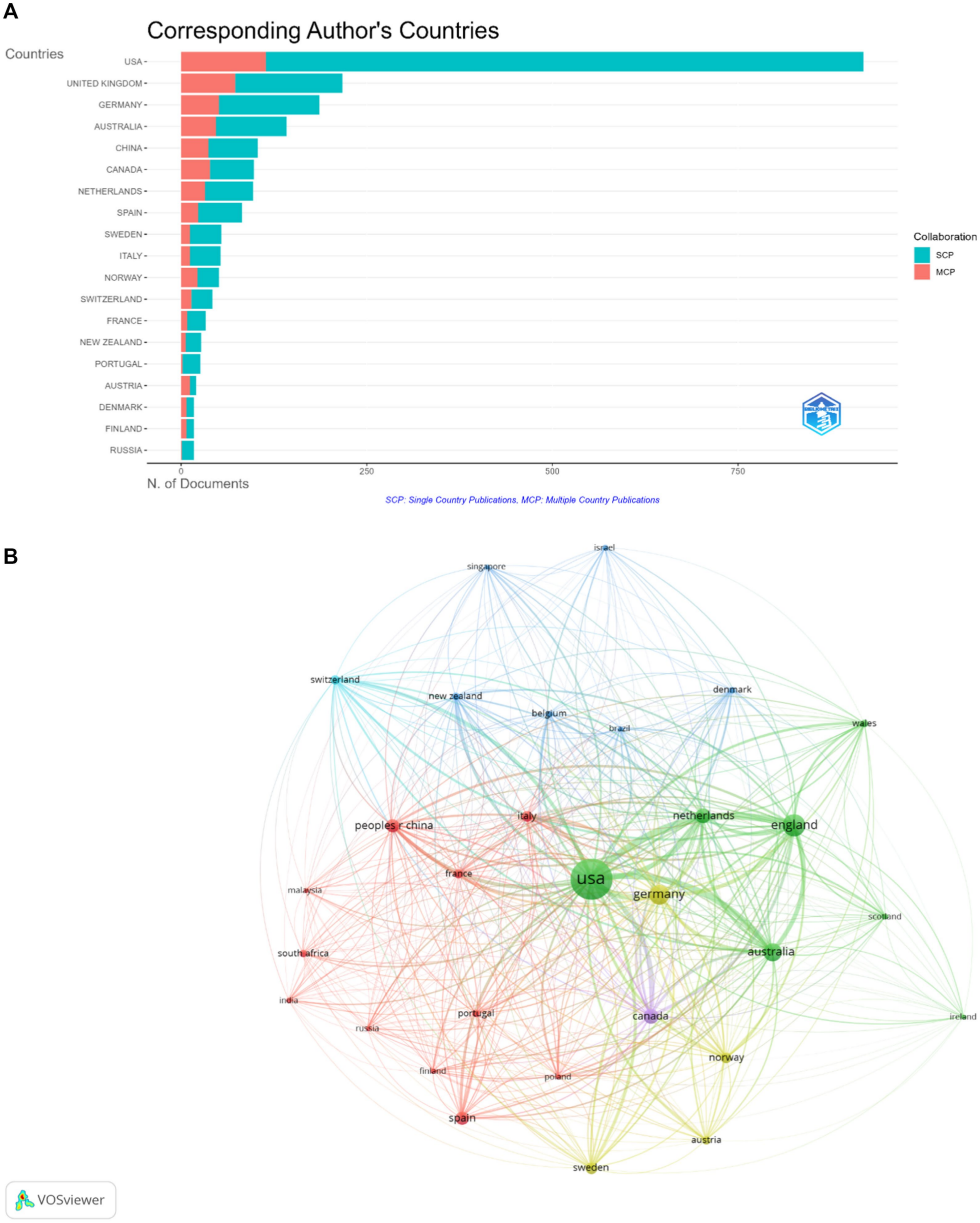
**(A)** Single country publications (SCP) and multiple country publications (MCP). Horizontal bar graph depicting the distribution of scientific documents by corresponding authors’ countries, classified by the type of collaboration. **(B)** Visualization of interconnectivity and interdependence of global research.

Figure 6A is a horizontal bar graph depicting the distribution of scientific documents by corresponding authors’ countries, classified by the type of collaboration: single country publications (SCP) and multiple country publications (MCP). The x-axis represents the number of documents, while the y-axis lists various countries. The USA leads substantially in both SCP and MCP, indicating a high level of both domestic and international collaborative research. The United Kingdom follows, with a significant number of SCPs and a relatively high number of MCPs, suggesting strong international collaborations as well. Germany, Australia, and China also show considerable numbers in both categories, underscoring their active roles in both national and international research arenas. Countries like Canada, the Netherlands, and Spain are also prominent on the list with both SCP and MCP, illustrating robust research environments within these countries in the Global North, both in domestic research and cross-border partnerships with each other. Figure 6A also shows that Sweden, Italy, Norway, and Switzerland have fewer documents but maintain a presence in international collaborations. France, New Zealand, and Portugal have a lower number of documents, with SCPs being more prevalent than MCPs, implying a stronger focus on national research efforts. Austria, Denmark, Finland, and Russia round out the list, with a smaller presence in the graph, indicating lesser but still significant academic contributions within this context. The figure suggests that the USA is the most prolific contributor to the field of study in question, followed by the UK and Germany. There is also a notable amount of international collaboration across most of the featured countries, as shown by the presence of MCPs.

The visualization in Figure 6B depicts a bibliometric network map illustrating the relationships between various countries based on academic collaborations. The nodes represent different countries, with the size of each node likely corresponding to the volume of research output or the number of citations that the country’s research has received. The lines between the nodes indicate collaborative relationships, with their thickness reflecting the extent or frequency of collaboration.

The USA is displayed as the largest node and central hub, suggesting it has the highest volume of research output or citations and is the most frequent collaborator with other countries. Other prominently sized nodes include Germany, England, and China, indicating these countries also have significant academic output and are key players in international research collaboration. Smaller nodes, like those representing Austria or Italy, while still visibly connected, indicate these countries have a more modest output or lower citation counts but are still active participants in the global research network. The multitude of lines connecting countries across the network signifies a robust international collaboration pattern, crucial for advancing global knowledge and scientific discovery.

The visualization underscores the interconnectivity and interdependence of global research efforts, with certain countries leading in output and collaboration, facilitating a diverse and integrated international academic community. This distribution reflects a skewed contribution from the Global North and an underrepresentation of the Global South despite the clear importance of both expertise and international cooperation in advancing scientific knowledge across the Global South.

### Citation and reference analyses

**Figure 7:**
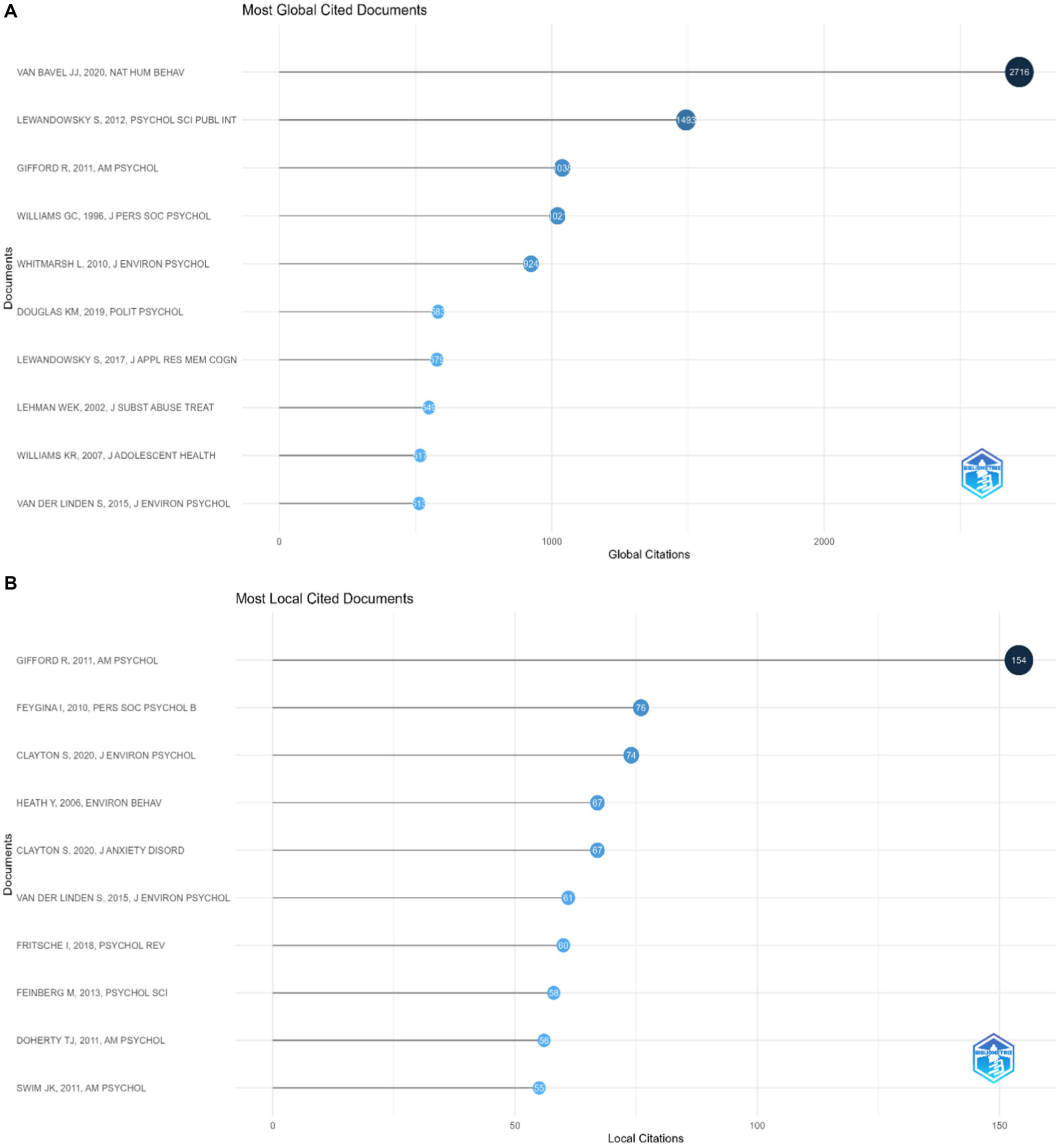
**(A)** Bar graph detailing scholarly documents that have garnered significant citation numbers on a global scale. **(B)** Local citation impact of various scholarly works.

In the next analysis we explored citations and reference analysis. Figure 7A, “Most Global Cited Documents,” features a bar graph detailing scholarly documents that have garnered significant citation numbers on a global scale. The document authored by Van Bavel JJ, published in “Nature Human Behaviour” in 2020, stands out with 2,716 global citations. This is followed by a work by Lewandowsky S., from 2012 with a significant citation count of 1,493. Other documents listed, by Gifford R., Williams GC., and Whitmarsh L., also show significant global citation counts, underscoring the relevance and reach of their contributions to the field.

The next analysis based on “Most Local Cited Documents.” Figure 7B presents a similar bar graph but focuses on the local citation impact of various scholarly works. Gifford R.’s 2011 paper in “American Psychologist” leads this chart with 154 local citations, highlighting its prominent role in specific scholarly communities or research areas. Following are works by Feygina I., Clayton S., and Heathy Y., all have a significant number of local citations, reflecting their impact within more localized and academic spheres. In the context of our previous analysis, these images provide insight into the dissemination and influence of research findings. They reveal which documents are shaping global academic discussions and which hold sway in more localized and academic contexts. This distinction is key for researchers who are looking to understand the reach of their work, identify key literature in their field, and grasp the landscape of international and local scholarly influence.

### Analysis of keywords

**Figure 8:**
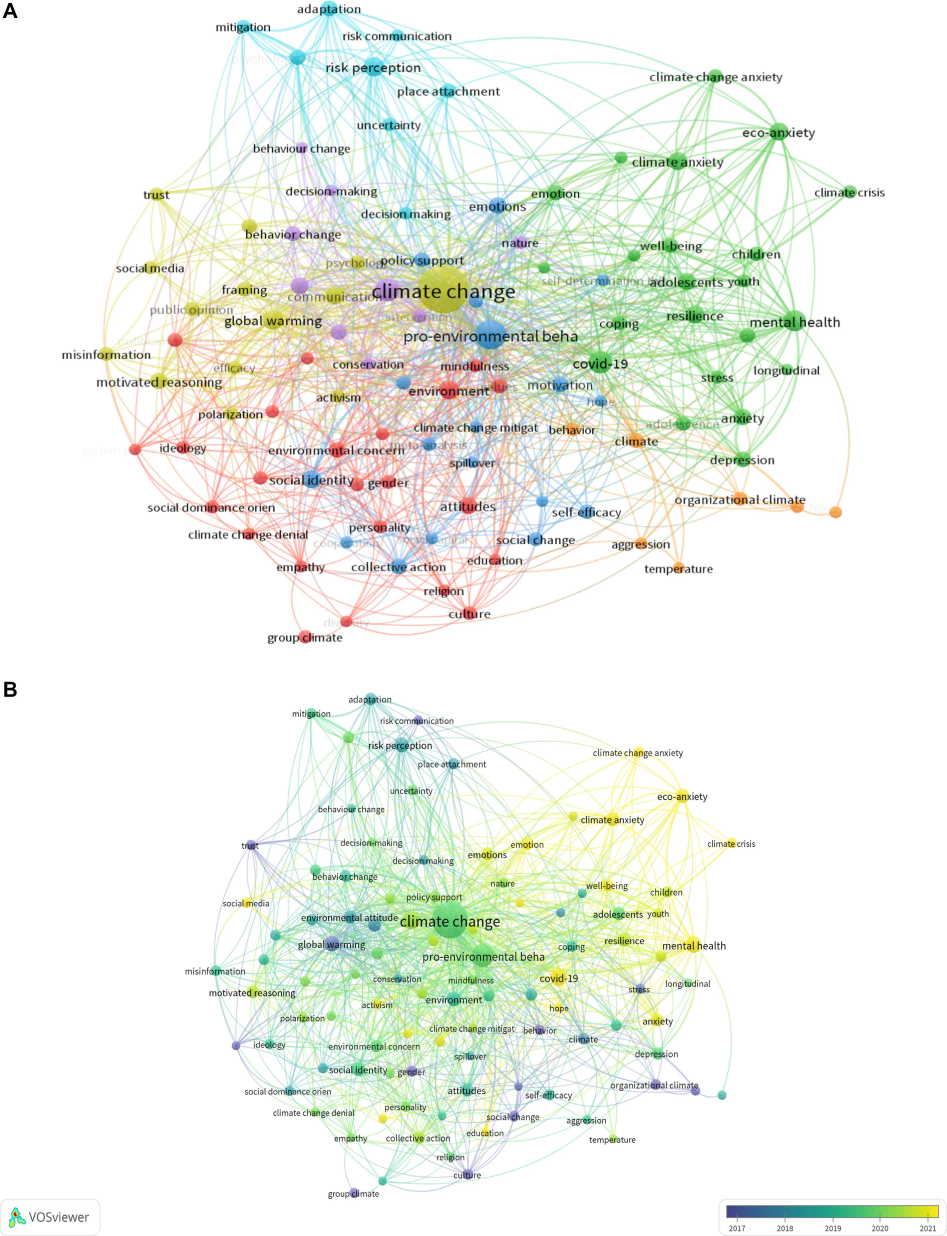
**(A)** A network visualization for the relationships between different terms within the body of literature. **(B)** Average publication year of the identified keywords.

We analyzed a total of 105 keywords among 5,781 keywords related to climate change on psychological topics that were identified as having occurred more than 10 times (Figure 8A). The colors in the overlay visualization shown in Figure 8B indicate the average publication year of the identified keywords. Most of the keywords were published after 2014, with greener or yellower colors.

Figure 8A is a network visualization commonly used in bibliometric analysis to map out the relationships between different terms within a body of literature. In this case, the visualization likely represents keywords or topics from research documents related to climate change and associated psychological aspects. The central and most prominent node is “climate change,” indicating that it’s the most frequently occurring term within the dataset and likely the primary focus of the research literature analyzed. Surrounding “climate change” are nodes representing related concepts such as “global warming,” “pro-environmental behavior,” “mental health,” and “eco-anxiety,” which suggests these topics are often discussed in conjunction with climate change. Other terms such as “COVID-19,” “risk perception,” and “social identity” are also part of the network, which implies a multi-disciplinary approach to the study of climate change, including its effects on human behavior, societal attitudes, and mental health challenges during overlapping crises, such as the COVID-19 pandemic. The various colors and clusters of nodes likely represent related themes or subtopics, suggesting areas of research that are often connected or that have emerged as significant subfields within the broader topic of climate change. The lines connecting the nodes indicate the relationships between these topics, with thicker lines representing stronger or more frequent associations between terms.

This network visualization serves as a tool for researchers to identify the most prominent topics in climate change-related research and how they intersect, as well as to identify emerging trends and potential gaps in the literature that might warrant further investigation. It shows the complex relationship between climate change, environmental issues and psycho-social dimensions. The colors in the overlay visualization shown in Figure 8B indicate the average publication year of the identified keywords. Most of the keywords were published after 2020, with greener or yellower colors are more recent one, indicating the recently trending topics in psychology of climate change.

Table 2 provided keywords and their frequencies of occurrence in the analyzed body of literature. “Climate change” is the most prevalent term with 567 mentions, which could reflect its status as a central topic within environmental research and academic discourse in psychology. The term “pro-environmental behavior” follows with 151 occurrences, indicating significant academic interest in how individuals can contribute to environmental sustainability, which itself is mentioned 68 times. The appearance of “COVID-19” 63 times suggests an overlap or an emerging area of study between the pandemic and environmental or climate studies, possibly exploring the intersections of public health and environmental issues. The inclusion of “mental health” (57 occurrences) alongside “global warming” (46 occurrences) and “climate change” suggests a research nexus where psychological aspects of climate issues are being examined. “Risk perception” and “environmental psychology” also appear, with 44 and 42 mentions respectively, pointing toward research into how climate issues are perceived as risks and their influence on educational environments.

**Table 2:** Keywords and their frequencies of occurrence in a corpus of literature on the psychology of climate change.

Words	Occurrences
Climate change	567
Pro-environmental behavior	151
Sustainability	68
COVID-19	63
Mental health	57
Global warming	46
Risk perception	44
Environmental psychology	42
Environment	39
Eco-anxiety	37
Social identity	36
Climate anxiety	34
Resilience	33
Communication	31
Adolescents	28
Attitudes	28
Environmental attitudes	28
Emotions	27
Anxiety	26
Motivated reasoning	26

The terms “eco-anxiety,” “social identity,” and “climate anxiety” with 37, 36, and 34 occurrences, respectively, imply a focus on the emotional and psychological responses to environmental challenges. “Resilience” and “communication,” mentioned 33 and 31 times respectively, suggest the investigation of how societies respond to and communicate about environmental stressors. The presence of “adolescents,” “attitudes,” and “environmental attitudes,” each mentioned 28 times, indicates a particular interest in the environmental perceptions of younger populations. “Emotions” (27 occurrences), “anxiety” (26 occurrences), and “motivated reasoning” (26 occurrences) further emphasize the psychological dimensions being explored in relation to environmental issues.

## Discussion

Climate change is an urgent global crisis affecting our ecosystems, human livelihoods, wellbeing, and social structures. Understanding this crisis requires a critical approach to understanding the landscape of the field, integrating insights from psychology, climate science, and social justice. This review uses a bibliometric analysis to map the global research landscape of the psychology of climate change and climate justice, identifying key contributions, thematic areas, and collaborative networks that shape this field. In the present study, we conducted a comprehensive bibliometric analysis supplemented by network visualizations to outline the evolving scholarship in the field of the psychology of climate change. We have examined this landscape of knowledge through the lens of critical psychology. This investigation cataloged the seminal contributions of authors, institutions, journals, and nations most of which are bases in the Global North. Furthermore, it shows the thematic trajectories anticipated to capture research interest in the forthcoming years. Bibliometric methodologies, recognized for their robustness and systematic approach, facilitate the exploration and quantitative evaluation of extensive scientific research.

Our application of bibliometric techniques to the selected databases shows the current research zeitgeist within this specialized field vis-à-vis both numerical data and graphical representations. It also spots the directions of new knowledge landscape indicating toward nascent research frontiers. Our findings show that the intersection of climate change psychology and critical psychology is garnering growing scholarly attention, reflecting an acknowledgment of their intersection significance in addressing climate-related challenges. The analysis shows the significance of relationships between human psychological processes and climate change, underscoring the need for nuanced understanding for example, inclusion of research and collaborations with the Global South (Anjum and Aziz, 2024), policy formulation, and intervention strategies. This study, therefore, serves as a pivotal step in synthesizing existing contributions while also identifying avenues for future inquiry within the critical psychology perspective on climate change.

Our bibliometric analysis has underscored the United States’ dominance in both national and international collaborative research, as evidenced by its substantial lead in both single country publication (SCP) and multiple country publications (MCP) metrics. This prominence is indicative of a robust research infrastructure needed for fostering significant domestic and cross-border academic endeavors. The United Kingdom and Germany also emerge as critical nodes within this scholarly network, demonstrating a strong inclination toward international collaboration, as reflected by their notable performances in SCP and MCP distributions. It is notable that most of these collaborations are still North to North collaborations and not as much focused on the Global South which might be indicative of the lack of infrastructure and other support networks in those regions.

The analysis further reveals a diverse spectrum of contributions from countries like Australia, China, Canada, the Netherlands, and Spain, highlighting a global commitment to advancing the understanding of psychological aspects of climate change. This diversity is essential for fostering a multifaceted understanding of climate change psychology, enriching the field with a variety of cultural perspectives and methodological approaches. Interestingly, nations such as Sweden, Italy, Norway, and Switzerland, despite generating fewer documents, have maintained a consistent engagement in international collaborations. This pattern suggests an openness to cross-national partnerships, enriching the global dialogue on climate psychology with the Global North. Conversely, France, New Zealand, and Portugal, with a greater emphasis on SCPs, underscore the importance of national research priorities and the cultivation of domestic expertise in addressing the psychological dimensions of climate change.

However, it is crucial to note that these nations, while significantly contributing to global greenhouse gas emissions, often have the resources and infrastructure to mitigate the direct impacts of climate change on their populations. Conversely, nations that are disproportionately affected by climate change-such as Small Island Developing States (SIDS), and countries in Sub-Saharan Africa and South and Southeast Asia-are markedly underrepresented in the psychological literature concerning climate change. This underrepresentation is not merely a matter of academic interest and literature; it has profound implications for climate justice. As Adger et al. (2013) argue, understanding the social dimensions of climate change is crucial for formulating effective and equitable adaptation strategies. Similarly, it is important to expand epistemological diversity in knowledge (see Adams et al., 2015; Anjum and Aziz, 2024) in the field of climate psychology. The psychological impacts of climate change, including stress, anxiety, and displacement, disproportionately affect these vulnerable populations, yet their voices and experiences are often absent from the scholarly discourse (Clayton et al., 2021; Anjum and Fraser, 2021).

The principle of climate justice emphasizes the need for equitable treatment and involvement of all people in addressing climate change, including the distribution of benefits and burdens and ensuring participatory decision-making processes (Schlosberg, 2013). From a psychological perspective, integrating the experiences and perspectives of those most affected by climate change is essential for developing comprehensive and culturally sensitive interventions. Furthermore, it is critical for ensuring that adaptation and mitigation strategies are grounded in the lived realities of those most at risk (Doherty and Clayton, 2011).

The prominence of universities primarily from Western countries in the list may reflect broader systemic issues within academia, including disparities in funding, access to publishing platforms, and visibility (Hickel, 2017). This imbalance raises questions about the global inclusivity of academic discourse on climate change, potentially sidelining voices and perspectives from the Global South or marginalized communities that are disproportionately affected by climate issues (Roberts and Parks, 2007). High citation counts and voluminous publication records do not necessarily indicate the social or ecological relevance of the research, particularly in addressing the multifaceted challenges of climate justice. Critical scrutiny is needed to evaluate whether the research outputs from these leading institutions are contributing to actionable solutions that address the root causes of climate injustice and support the needs of vulnerable populations (Agyeman et al., 2003). The dominance of certain institutions in academic rankings can perpetuate power imbalances in the creation and dissemination of knowledge. Critical psychology encourages an examination of how institutional prestige influences which research questions are prioritized, whose knowledge is valued, and how findings are applied to address societal issues (Prilleltensky, 2003). This perspective invites a critical assessment of whether the research from these top-ranked universities challenges or reinforces existing social and psychological structures contributing to climate change and inequality. The impact of academic research in climate psychology should also be measured by its engagement with communities, including its contribution to public understanding, policy development, and community-based solutions to climate change (Cunsolo and Landman, 2017).

Evaluating the academic impact of universities through climate justice and critical psychology highlights the need for a more inclusive definition of impact. This approach emphasizes equity, relevance, and engagement with diverse communities. It advocates for inclusive academic publishing and discourse, ensuring that researchers from various backgrounds and regions have equal access to publishing, funding, and visibility. Moreover, it calls for interdisciplinary and collaborative research that bridges the gap between academia and society, addressing climate change and social justice directly. Encouraging scholars to reflect critically on how their work influences social and environmental justice is essential for fostering a more equitable academic landscape.

### Influential authors and work in the field of psychology and climate change

The three most cited authors and their most influential work are Sander Van der Linden, Susan Clayton, and Robert Gifford. These are discussed in the light of critical psychology. Sander van der Linden appeared as one of the most cited authors. His most cited publication in climate change perspective published in the Journal of Environmental Psychology was about a comprehensive model about the public’s perceptions of climate change risk by weaving together cognitive, experiential, and socio-cultural dimensions. From a critical psychology perspective, while the model integrates cognitive, experiential, and socio-cultural dimensions, it could further explore how societal structures and power dynamics influence these perceptions and the dissemination of misinformation. The focus on individual cognition and behavior change, without equally addressing the systemic misinformation campaigns by powerful industries, might limit the scope of intervention necessary for widespread climate action. This research underscores the need for a more holistic approach that considers the systemic origins of climate (mis)information and its impacts on public perception and behavior.

Susan Clayton appeared as the other most cited and prominent author in the field of psychology and climate change. Her most cited work in the Journal of Environmental Psychology in our document analysis was around climate change anxiety and how it affects cognitive and functional impairment factors and also with behavioral engagement. This highly cited work examines the concept of climate anxiety, presenting evidence for its existence and discussing potential approaches to address it. While acknowledging the importance of recognizing climate anxiety as a genuine concern deserving clinical attention, the paper emphasizes the need to differentiate between adaptive and maladaptive levels of anxiety. However, through the lens of critical psychology, this work can be expanded to consider how systemic and structural factors contribute to climate anxiety. The differentiation between adaptive and maladaptive anxiety, while clinically relevant, also invites a broader discussion on how societal norms, economic pressures, and political inaction exacerbate feelings of helplessness and anxiety among individuals. Recognizing climate anxiety not just as a personal concern but as a societal issue reflects the interconnectedness of individual wellbeing and systemic health, pointing toward the necessity of structural solutions to mitigate both climate change and its psychological impacts. This expansion is particularly relevant now because of the climate change related extreme events are unparalleled and happing at much larger scales which can be only addressed with systematic and structural solutions to climate anxiety.

Robert Gifford was also one of the most top cited researcher in the field. His most cited document is about psychological barriers to climate action, referred to as the “dragons of inaction,” include limited cognition about the problem, ideological worldviews, comparisons with others, sunk costs, discordance toward experts, perceived risks of change, and inadequate behavior change, highlighting the need for collaboration between psychologists, scientists, experts, and policymakers to address these challenges effectively. A critical psychology approach would extend this analysis to examine how these barriers are not only individual but are also embedded within and reinforced by societal norms, economic systems, and political structures that resist change. For instance, ideological worldviews and comparisons with others are shaped significantly by the cultural and media landscapes, while perceived risks of change and inadequate behavior change are influenced by economic systems that prioritize short-term gains over long-term sustainability. The emphasis on collaboration among psychologists, scientists, experts, and policymakers implicitly acknowledges the need for systemic change; however, critical psychology would argue for a more explicit focus on dismantling the structures that perpetuate inaction.

### Hot topics of psychological research on climate change

The study’s analysis of 105 most repeated keywords from a dataset of 5,781 words related to climate change on psychological topics indicates a growing academic focus on the psychological dimensions of climate change, particularly post-2014. This temporal trend suggests an increasing recognition of the importance of psychological research in understanding and addressing climate change. The prominence of terms like “climate change,” “pro-environmental behavior,” and “mental health” reflects a comprehensive exploration of how individuals perceive, are affected by, and can act upon climate-related issues. Notably, the appearance of “COVID-19” within the literature highlights the interconnectedness of global crises, pointing to a burgeoning area of research that examines the synergistic effects of the pandemic and climate change on public health and environmental sustainability. This intersection is particularly relevant for critical psychology, which seeks to understand how crises amplify existing social inequalities and impact mental health (Pfefferbaum and North, 2020; Clayton et al., 2015).

The focus on “eco-anxiety,” “social identity,” and “climate anxiety” underscores the emotional and psychological repercussions of climate change. This aligns with findings from environmental psychology that document the emotional burden of climate change and the importance of psychological resilience and adaptive coping strategies (Clayton, 2020; Doherty and Clayton, 2011). The bibliometric network visualization facilitates the identification of core and peripheral research themes, offering insights into the interrelatedness of different aspects of climate change psychology. This networked approach is crucial for understanding the complexity of the field and for identifying emergent research areas that require further exploration.

While the study provides valuable insights, it also invites critical reflection from the perspectives of critical psychology and climate justice. One critique pertains to the potential overemphasis on individual behavioral responses to climate change (“pro-environmental behavior”) without adequate consideration of the structural and systemic factors that constrain individual agency (Crompton and Kasser, 2009; Swim et al., 2011). Critical psychology emphasizes the importance of examining the socio-political contexts that shape environmental behaviors and attitudes, suggesting that future research should integrate analyses of power dynamics, social inequalities, and the role of institutions in facilitating or hindering climate action (Prilleltensky, 2012).

Moreover, the inclusion of “COVID-19” in climate change research presents an opportunity to critically examine how responses to the pandemic can inform more equitable and effective approaches to climate action. Climate justice frameworks emphasize the need to address the disproportionate impacts of climate change on marginalized communities and to ensure that responses do not exacerbate existing inequalities (Schlosberg, 2013; Agyeman et al., 2003). Future research could benefit from a more explicit focus on equity and justice in the context of overlapping crises.

Additionally, the study’s findings on “eco-anxiety” and “climate anxiety” highlight the emotional toll of climate change, but there is a need for further research that explores the differential impacts of these psychological responses across diverse populations and contexts. Climate justice perspectives would advocate for studies that examine how socio-economic status, race, ethnicity, and geographical location influence individuals’ emotional responses to climate change and their access to coping resources (Hickman et al., 2021; Anjum and Fraser, 2021).

Climate justice perspectives argue that framing climate change impacts in terms of individual psychological responses, such as “eco-anxiety” or “climate anxiety,” risks diverting attention from the systemic, structural causes of climate change and the uneven distribution of its impacts. This framing can overshadow the need for systemic change by focusing on individual emotional responses rather than addressing the root causes of environmental degradation and the disparities in who is most affected (Harlan et al., 2015). Climate justice advocates emphasize the importance of addressing the socio-economic and political dimensions of climate change that disproportionately affect marginalized communities (Schlosberg and Collins, 2014). Discussions around “eco-anxiety” and “climate anxiety” often emerge from a Western, middle-class perspective, potentially marginalizing the voices and experiences of those in the Global South or disadvantaged communities who are facing the most immediate and severe impacts of climate change. This critique highlights the need for inclusivity in the conversation about emotional responses to climate change, ensuring that it encompasses the diverse experiences and challenges faced by different populations globally (Norgaard, 2011).

Critical psychology critiques might argue that terms like “eco-anxiety” and “climate anxiety” pathologize natural and rational responses to the existential threat of climate change. This pathologizing could potentially medicalize and individualize what are essentially collective and societal issues, diverting attention from collective action toward individual therapy and coping mechanisms. Pihkala (2020) suggests that while acknowledging these anxieties is important, it is equally crucial to frame them in a way that mobilizes action and acknowledges their root in rational concerns about the planet’s future.

Critical psychology also points to the potential overemphasis on emotional responses at the expense of understanding and tackling the cognitive, social, and political dimensions of climate change. This includes how societal structures, norms, and values contribute to environmental degradation and how these can be challenged and changed. There is a call for a more holistic approach that includes but is not limited to, addressing emotional responses, fostering critical consciousness, and promoting social and political engagement (Fisher, 2013).

### Current trends

The study reveals an increasing and current academic interest in the emotional and psychological responses to climate change, particularly “climate anxiety” and “eco-anxiety,” alongside the broader category of “mental health.” This trend is indicative of a growing recognition of the psychological dimensions of climate change, not just as peripheral concerns but as integral aspects of the climate crisis that require attention and action.

The prominence of terms like “climate anxiety” and “mental health” in the literature signifies an acknowledgment of the complex ways in which climate change affects individuals’ psychological wellbeing. This aligns with recent research suggesting that awareness of and concern for climate change can lead to significant emotional distress, anxiety, and feelings of helplessness (Clayton, 2020; Cunsolo and Ellis, 2018). The discourse around “climate anxiety” and “eco-anxiety” is increasingly being understood as a rational response to the existential threat posed by climate change. This perspective challenges traditional notions of anxiety as pathological, framing these emotional responses instead as appropriate reactions to the scale and scope of the environmental crisis (Pihkala, 2020). The recognition of mental health impacts necessitates a broader public health and policy response that goes beyond mitigation and adaptation strategies to address climate change. It calls for integrating mental health support into climate action plans and ensuring that communities, particularly those most vulnerable to climate change, have access to psychological resources (Doherty and Clayton, 2011; Hayes et al., 2018).

### Strategies for equitable collaboration

Strengthening local and regional scholarly networks, valuing and validating publication outlets in the global South, and establishing clear collaboration agreements are vital strategies for fostering equitable collaboration between scholars from the Global North and South (Anjum and Aziz, 2024; Teferra and Altbachl, 2004). Local mentorship and training within the Global South are essential for nurturing future scholars and ensuring research remains culturally pertinent and methodologically sound (Manuh et al., 2007; Nyamnjoh, 2012). For collaborators from the Global North, genuine collaboration rooted in trust, mutual respect, and recognition of all partners’ expertise and local knowledge is crucial (Anjum and Aziz, 2024). These efforts should include mutual capacity building, recognizing indigenous methodologies, and ensuring fair representation in publications (Tervalon and Murray-García, 1998; Hall and Tandon, 2017).

### Limitations and future directions

We acknowledge that limiting our analysis to English-language publications introduces a methodological bias. This reflects the dominance of English in international academic discourse but may overlook critical work published in other languages, particularly from regions such as Latin America, Asia, and Africa. Future analyses should incorporate regional databases and multilingual bibliometric tools to capture diverse perspectives. There are non-English journals, particularly in Latin America, Asia, and Africa, that address climate psychology topics and remain underrepresented. Identifying and incorporating these sources in future work will help mitigate existing biases and better reflect diverse scholarship.

Another important limitation of this study is the lack of analysis of the geographical focus, methodological diversity, and epistemological approaches of the studies identified in our bibliometric analysis. While this study primarily aimed to map thematic and network patterns, a more detailed investigation into where studies are conducted versus where they are authored would provide valuable insights into the dynamics of “climate colonialism,” a phenomenon where research from the Global North often focuses on vulnerable communities in the Global South without adequately addressing systemic inequalities. Future research should explore these dimensions to uncover the implications of Global North dominance in shaping research agendas, methodologies, and narratives in climate psychology. Such an approach could offer a richer understanding of how research practices perpetuate or challenge existing inequities and contribute to building a more inclusive and representative body of scholarship. It is crucial to distinguish between where studies are empirically conducted and where they are authored. Often, research from the Global North focuses on Global South communities without adequate representation of scholars from these regions.

## Conclusion

This comprehensive bibliometric analysis has indicated what are significant trends and contributions within the field of psychology and climate change, covering research from 1995 to 2024. This study has thoroughly explored scholarly output, employing critical psychology perspectives, and has particularly focused on the emergent concept of research equity and climate justice. By mapping the intellectual landscape, our analysis has identified key authors, collaborative networks, and thematic focuses that characterize this dynamic field. Notably, authors and scholarship from nations like the United States, United Kingdom, and Germany are prominent due to their robust research infrastructures. This also facilitates significant scholarly output and international collaboration within the countries in the Global North. However, a notable gap exists in the representation from regions most affected by climate change, such as Small Island Developing States (SIDS) and countries in Sub-Saharan Africa, and South and Southeast Asia. This underrepresentation underscores an urgent need for a more inclusive global research agenda that not only tackles scientific questions within climate psychology but also integrates socio-economic and cultural dimensions that influence vulnerability to climate impacts.

In terms of thematic focus, our bibliometric study has highlighted a strong emphasis on pro-environmental behavior, mental health, and the nuanced exploration of climate anxiety and eco-anxiety. These topics emphasize the psychological toll of climate change and underscore the necessity of incorporating mental health support within comprehensive climate action plans. By categorizing these psychological impacts as rational responses to environmental crises, our findings advocate for the development of policy frameworks that address both the ecological and emotional dimensions of climate change. Furthermore, the dominance of Western institutions in our findings prompts a critical reflection on current academic discourse, which often prioritizes methodologies and perspectives that might not fully capture the complexities of global climate impacts especially for the Global South. This highlights the need for a more equitable approach to knowledge creation, co-production, and dissemination, ensuring that diverse perspectives and experiences are integrated into future work on climate research and action.

In order to bridge the gap between empirical research and practical application, it is crucial to engage directly with affected communities, policymakers, and practitioners. This engagement will enhance the relevance and impact of our research, ensuring that it contributes to academic knowledge and facilitates real-world change. Our study calls for an interdisciplinary and collaborative approach that transcends traditional academic boundaries, aiming to challenge systemic barriers to climate action and contribute actively to sustainable and just solutions for global climate challenges. Through its rigorous bibliometric approach and critical analysis, this research has laid the groundwork for future scientifically sound and socially equitable endeavors to make a significant impact in the ongoing global dialogue on climate change and psychological resilience.

## Data Availability

The original contributions presented in the study are included in the article/supplementary material, further inquiries can be directed to the corresponding author.

## Footnotes

^1^ https://osf.io/m6tay/?view_only=5bf1760d08ef44709e8d8dfdaae47d94

^2^ https://www.bibliometrix.org
